# Impact of tumor necrosis factor-alpha gene variant in pediatric nephrotic syndrome: a meta-analysis

**DOI:** 10.1038/s41598-025-15387-w

**Published:** 2025-08-14

**Authors:** Yogalakshmi Venkatachalapathy, Praveenkumar Kochuthakidiyel Suresh, Thendral Hepsibha Balraj, Vettriselvi Venkatesan, Sangeetha Geminiganesan, Indira Bhagam, C. D. Mohana Priya

**Affiliations:** 1 Department of Human Genetics, Sri Ramachandra Institute of Higher Education and Research, Chennai, India; 2 Department of Biochemistry, Ethiraj College for Women, Chennai, India; 3 Kauvery Hospital, Chennai, India; 4 Sri Ramachandra Institute of Higher Education and Research, Chennai, India

**Keywords:** Childhood nephrotic syndrome, Meta-analysis, *TNF*-α polymorphism, Pediatrics, Genetic variant, Molecular biology, Risk factors

## Abstract

This study aims to explore the risk associated with the Tumour Necrosis Factor-Alpha (TNFα)-308 G/A (rs1800629) gene polymorphism in relation to Childhood Nephrotic Syndrome (NS). The primary goal of the meta-analysis was to investigate the relationship between Nephrotic Syndrome and the TNFα 308 G/A [rs1800629] polymorphism of the cytokine gene. We conducted a systematic search across electronic databases like PubMed and Google Scholar to collect data from five distinct case-control studies focused on the *TNF*α 308 G/A gene variant, covering the period from 2010 to 2022. By aggregating allele and genotype frequencies from these studies, we computed the 95% confidence interval of odds ratio [OR] to assess the strength of the association. To evaluate heterogeneity and potential publication bias in the selected studies, Stats Direct software was employed. The sample size encompassed 1,560 individuals, including 628 cases and 932 controls from five separate case-control studies. The *TNF*α allele-A displayed significant heterogeneity [I²=80%, 95% CI] when compared to the G allele, and a statistically significant pooled OR of 2.32 [*P* = 0.0056*] was observed. In the overall analysis, no significance association was found in the dominant model [*P* = 0.068], but a significant association was detected in the recessive model [*P* = 0.0096*]. Consistent findings were observed in the co-dominant model, where both AA vs. GG [*P* = 0.0075*] and GA vs. GG [*P* = 0.0219*] showed significant associations. These results suggest a potential increase in the risk of the disease associated with the polymorphism of *TNF*α 308 G/A. The small number of studies, high heterogeneity (I² = 80%), and limited ethnic diversity may affect the robustness and generalizability of findings. Additionally, language bias and lack of confounder adjustment limit interpretability. The *TNF*α 308 G/A polymorphism was identified as being associated with the risk of developing childhood NS.

Registration: Registered in PROSPERO with ID: CRD420251083425.

**Supplementary Information:**

The online version contains supplementary material available at 10.1038/s41598-025-15387-w.

## Introduction

Nephrotic syndrome (NS) is characterized by marked proteinuria, low levels of albumin in the blood, and the development of edema. It is estimated that NS affects roughly 1–3 per 100,000 children who are below 16 years old. Remarkably, about 85% of individuals with NS experience a full resolution of proteinuria when treated with standard doses of daily oral prednisolone^1,2^. The assessment of the complexity and prognosis of this syndrome is determined by a combination of how well it responds to steroids and the accompanying histological findings. This specific category is referred to as steroid-sensitive nephrotic syndrome (SSNS). If individuals fail to attain remission following 4–6 weeks of steroid treatment, they are typically to be at a heightened risk of developing steroid-resistant nephrotic syndrome (SRNS). The predominant histopathological patterns observed in cases of SRNS primarily involve focal and segmental glomerulosclerosis (FSGS), with a smaller proportion showing minimal change disease (MCD) and diffuse mesangial sclerosis^3^. New research has indicated that the development of nephrotic syndrome is quite intricate. There is some evidence to indicate that NS may be a primary immunological disorder in which the primary malfunction of Thymus-cells [T-Cell] is linked to an immuno-regulatory Disparity in T helper subtype 1 & 2 (Th1,Th2). The precise mechanisms behind this imbalance remain unclear. The pathophysiology of NS still lacks clarity, and it appears that a range of inflammatory cytokines, chemotactic substances, and transcription factors play a role in the disease’s progression and its related symptoms^4,5^. Th1 and Th2 cells secrete cytokines, which encompass Interleukin (*IL*)- *2*, *IL-4*,* IL-5*,* IL-6*,* IL-10* and *IL-13*^6^. It has been documented that the origin and progression of the disease are affected by substances released into the bloodstream by these activated T-cells. Th1 cytokines are pivotal in the advancement of Focal Segmental Glomerulosclerosis (FSGS), whereas cytokines are significant factors in Minimal Change Nephropathy (MCN). These conditions represent the leading culprits behind the development of NS in both children and adults^7^. One of these elements, TNF-alpha, a powerful immune regulator and proinflammatory cytokine, has been associated with the development of several medical conditions^8^. TNF-alpha has exhibited connections with various inflammatory disorders, such as glomerulonephritis, ankylosing spondylitis, and multiple sclerosis^9–11^. Functional single nucleotide polymorphisms (SNPs) of TNF-α cytokine genes located in the promoter region have been discovered to impact both the function of the gene promoter and the levels of gene product production^12,13^. A variant at position 308 G/A in the TNF-α promoter linked to increased transcription of TNF. An earlier study has shown an increase in the production and expression of TNF-α in individuals with primary NS^14^. The choice of these particular genes is based on the potential for single nucleotide variant within these genes to influence an individual’s vulnerability to the disease or modify the clinical course of the disease. The SNP TNFα -308 G/A (rs1800629) is located on chromosome 6 at position 6p21.3, within the promoter region of the TNF gene.The current research focuses on analyzing genetic variants in the TNFα cytokine gene at position 308 G/A in both children with NS and a control group of healthy individuals. Many previous studies have examine the connection between TNFα 308 G/A polymorphisms and childhood Nephrotic syndrome in various populations^7,15–18^. Hence, the indispensability of this meta-analysis is to validate the correlation between the TNFα 308 G/A [rs1800629] genetic polymorphism in the cytokine gene and Nephrotic Syndrome in children. This analysis is aimed at providing a improved insight of the involvement of inflammatory marker in NS.

## Methods

### Literature search

We utilized electronic databases such as PubMed and Google Scholar to conduct a thorough search for case-control studies that examined the connection between the TNFα 308 G/A gene polymorphism and the probability of childhood Nephrotic syndrome. The selection process for including studies in the meta-analysis was carried out meticulously using the database, and a summary of this process is outlined in Table 1. We collected research articles published between 2010 and 2022 for evaluation. To perform the literature search, we employed a combination of keywords, including terms like TNFα or Cytokine genes, inflammatory markers or genes, Tumour Necrosis Factor-alpha, gene or allele or polymorphism, interleukin gene, and childhood Nephrotic Syndrome. We scrutinized the title, abstract, and references of all relevant publications carefully. Whenever feasible, full-text articles from these relevant sources were chosen and integrated into the analysis. The statistical software Stats Direct [version 3.0] was employed to compute the pooled Odd’s Ratio(OR) with a 95% CI. The study was approved by the Institutional Ethics Committee of the Sri Ramachandra Institute of Higher Education and Research (SRIHER) in Porur, Chennai, India, under reference number IEC-NI/22/JUL/83/71.

**Table 1:** Main characteristics of the eligible studies included in this meta-analysis.

SNP	First author	Year	Ethnicity	Gender		Mean age		Genotyping method	Sample size		HWE P-value
				Case (M/F)	Control (M/F)	Case	Control		Case	Control	
*TNFα* *308 G/A* *(rs1800629)*	Simin Sadeghi-Bojd	2021	Iran	96 / 72	74 / 79	5.77 ± 3.01	6.03 ± 2.3	PCR-RFLP	168	153	0.36
	DoaaM. Youssef	2017	Egypt	76/74	71/79	4.4 ± 1.8	4.7 ± 1.9	PCR-RFLP	150	150	0.90
	Tabrez Jafar	2011	India	106/44	399/170	11.0 ± 6.6	12.0 ± 3.5	PCR-RFLP	150	569	0.81
	Dina Abdel Razek Midan	2017	Egypt	63/37	19/11	7.51 ± 3.12	8.47 ± 3.80	PCR-RFLP	100	30	0.62
	Hanan A. Madani	2014	Egypt	31/29	20/10	6.19	6.44	PCR-RFLP	60	30	0.06

### The selection process for research articles in this meta-analysis involved clearly delineated requirements

The eligibility conditions were as follows:

- Case-control studies conducted between 2010 and 2022.
- Studies that evaluated the risk associated with TNFα -308 G/A gene polymorphisms in childhood Nephrotic syndrome.
- Availability of adequate data for calculating the OR along with a 95% CI.
-Genotype frequencies in the control groups of all included studies were assessed for conformity with Hardy-Weinberg Equilibrium (HWE) using the chi-square (χ²) test. and by a P-value > 0.05.

The specific elimination conditions are:

- Studies that did not have control groups.
- Exclusion of research involving experimental models involving animals, those absence of genotype information, summaries, case analyses and comprehensive reviews.
- Studies in which genotype distributions in the control group significantly deviated from HWE (*P* ≤ 0.05).

### The extraction of data and evaluation of study quality for the selected research

The researcher, Yogalakshmi, conducted a comprehensive examination and data extraction, which included details like the author’s profile, article type, year of publication, study population, sample size for both cases and controls, type of polymorphism, and genotyping data. To evaluate the methodological attribute of the studies incorporated in the meta-analysis, the Newcastle-Ottawa Scale [NOS] was employed. The NOS employs a star-based rating system, encompassing three key categories: the selection of study participants [both cases and controls], the comparability of these participants, and the ascertainment of exposure. The highest possible score on the NOS scale is 9 points, with a score of ≥ 7 points indicating a study of high quality.

## Data analysis

95% confidence intervals (CI) were used to assess the strength of the association between the TNFα − 308 G/A genetic polymorphism and childhood Nephrotic Syndrome. Statistical significance was defined as a P-value of less than 0.05. Cochran’s Q-test was used to assess whether there was variation among the combined studies with respect to the gene variant, and the I^2^ statistic was used to measure the degree of heterogeneity.

For every genetic comparison model, 95% Prediction Intervals (PIs) were computed in addition to I^2^ in order to estimate the anticipated range of effect sizes in subsequent research. The 95% PI takes into consideration between-study heterogeneity and gives a range that the true effect size of a new study is likely to fall within, in contrast to the 95% CI, which represents the precision of the pooled estimate.

Heterogeneity was classified into four categories: low, moderate, large, and extreme, based on the I^2^statistic, with ranges of 0–25%, 25–50%, 50–75%, and > 75%, respectively. In cases of low heterogeneity (I² ≤ 25%), the pooled odds ratio (OR) was computed using the Mantel-Haenszel method with a fixed-effects model, whereas in cases of moderate to high heterogeneity (I² > 25%), the DerSimonian-Laird method with a random-effects model was applied.

To evaluate the likelihood of publication bias, we performed a Begg’s Funnel plot analysis. Any irregularities observed in the Begg’s funnel plot were further assessed using Egger’s regression test, where a P-value less than 0.05 would suggest the potential existence of statistically significant publication bias.

### Literature selection and study subject characteristics

**Fig. 1:**
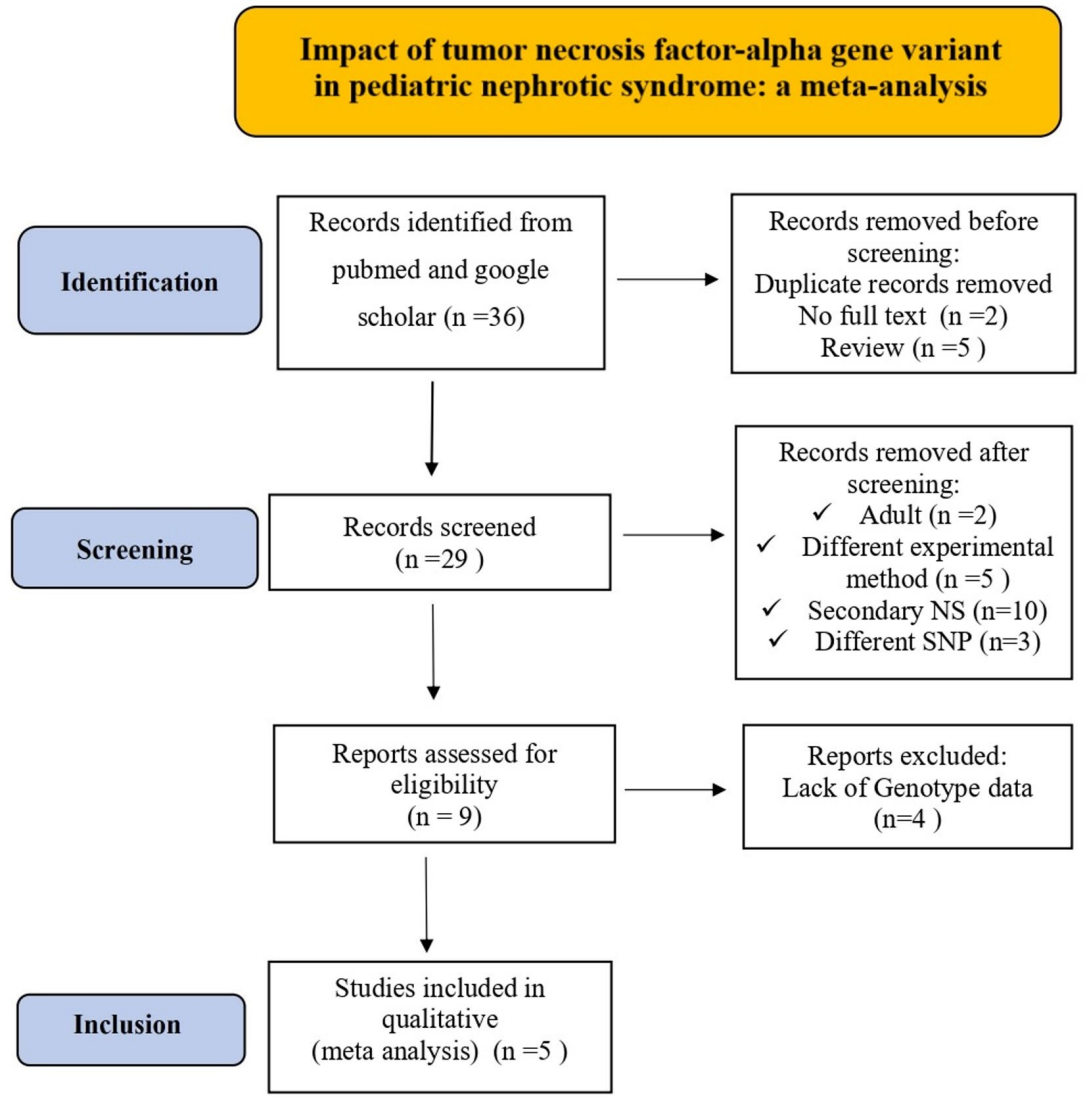
PRISMA flow diagram of studies selected for the meta-analysis.

The process of selecting research articles for the meta-analysis on TNFα polymorphisms in childhood NS followed the principles laid out in the PRISMA (Preferred Reporting Items for Systematic Reviews and Meta-Analyses) framework^19^ represented in Fig. 1. At the outset, 36 studies were initially identified through a database search. After eliminating those without full-length articles and review records, 29 relevant studies remained. During the screening process, 20 studies were excluded for various reasons, including differences in study design, the focus on adult NS, studies examining the same gene but with different SNPs, and studies related to secondary NS.

Additionally, four publications were excluded because they either lacked genotyping data or investigated the association of different SNPs within the selected genes considered for this meta-analysis. As a result, a sum of five suitable studies that met the established inclusion and exclusion criteria were selected to be part of this current study. The particulars of these selected studies employed in the analysis are outlined in Table 1.

Altogether, the encompassed studies encompassed 628 instances and 932 individuals serving as controls. Upon scrutinizing the methodological quality of these five studies, it was observed that the NOS scores varied from 7 to 9, yielding an average score of 8.4 points^7,15–18^. These combined scores signify a high level of quality in the chosen literature. The NOS scores for each of the selected studies can be found summarized in Table 2.

**Table 2:** Newcastle Ottawa (NOS) scale for assessing the quality of the selected case-control study.

First author & year	Selection				Comparability		Exposure			Total score
	a	b	c	d	e	f	g	h	i	
Simin et al.,2021	*	*	*	*	*	**	*	*	-	9
Doaa et al.,2017	*	*	*	*	*	*	*	*	-	8
Tabrez et al.,2011	*	*	*	*	*	**	-	*	-	7
Dina et al.,2017	*	*	*	*	*	**	*	*	-	9
Hanan et al.,2014	*	*	*	*	*	**	*	*	-	9

Quality assessment checklist

a. Is the case definition adequate?

b. Representativeness of the Cases.

c. Selection of Controls.

d. definition of controls.

e. Study controls for ethnicity.

f. Study controls for any additional factor.

g.Ascertainment of exposure.

h.The same method of ascertainment for cases and controls.

i.Non-response rate.

### Outcomes/result

A meta-analysis of cytokine gene polymorphism and childhood Nephrotic Syndrome was conducted, focusing on the TNFα − 308 G/A variant. A total of 628 cases and 932 controls were included across five case-control studies. The analysis revealed considerable heterogeneity (I² = 80%) between the TNFα allele-A and G allele. Specifically, allele-A showed a statistically significant pooled OR of 2.32 (95% CI: 1.27–4.20; *P* = 0.0056*), with a **95% PI of 1.00–5.37**, indicating a potentially strong association.

Among genetic models, the dominant model (GA + AA vs. GG) showed a pooled OR of 2.57 (95% CI: 1.29–5.12; *P* = 0.0068*) with I² = 79.6% and a 95% PI of 0.97–6.81, suggesting possible variability. The recessive model (AA vs. GA + GG) showed a significant association with OR = 5.05 (95% CI: 1.48–17.23; *P* = 0.0096*), I² = 33%, and a 95% PI of 0.89–28.65.

For the co-dominant model, the GA vs. GG comparison yielded OR = 1.86 (95% CI: 1.09–3.17; *P* = 0.0219*), I² = 63.7%, and 95% PI: 0.88–3.95. The AA vs. GG comparison showed OR = 5.69 (95% CI: 1.59–20.36; *P* = 0.0075*), I² = 37.4%, and 95% PI: 0.94–34.52.

**Fig. 2:**
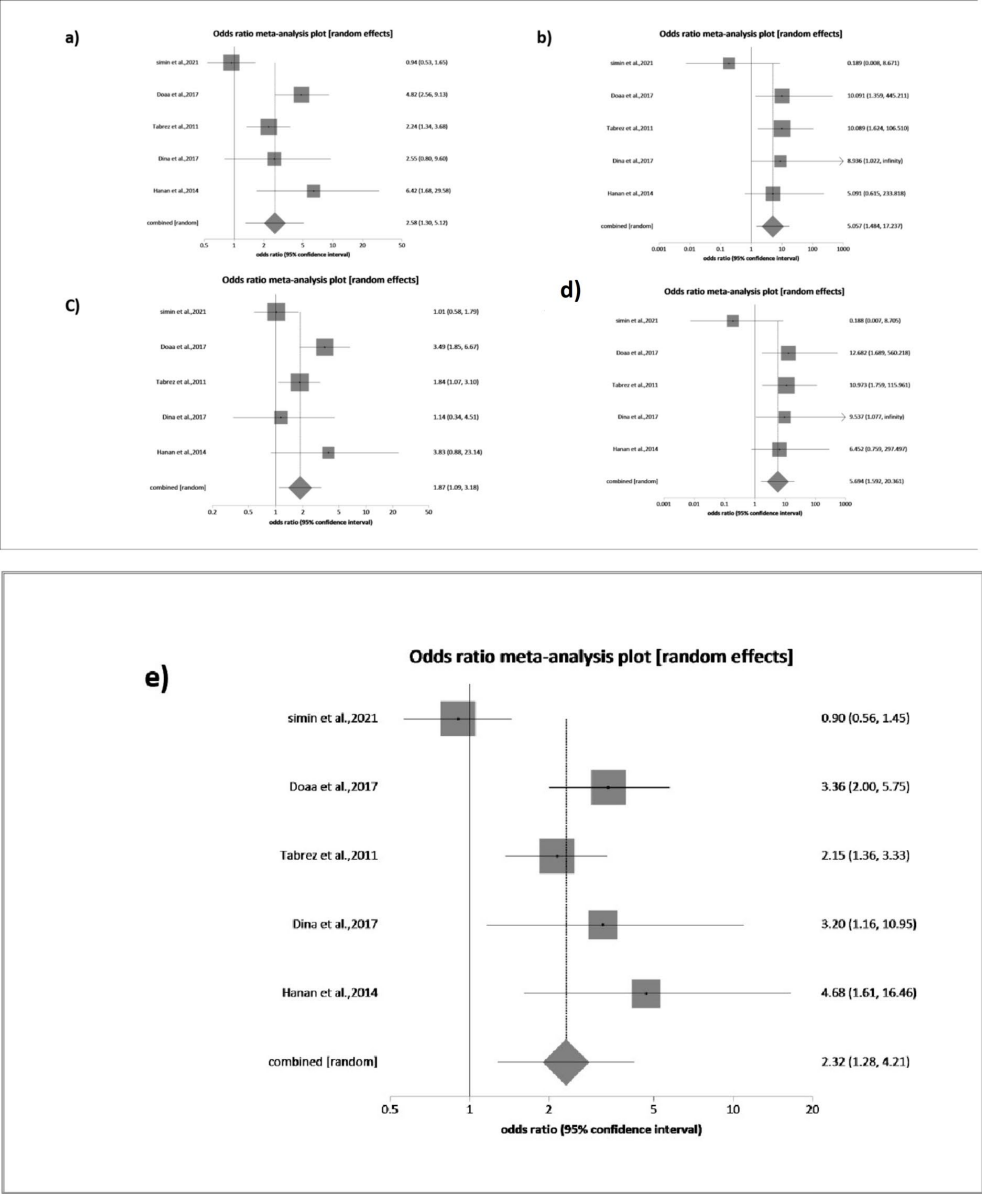
Forest plot for the meta-analysis of (*TNFα 308 G/A*) polymorphism associated with NS risk. (a) Dominant model, (b) recessive model, (c) GA vs. GG, (d) AA vs. GG, and (e) A vs. G allele. OR = Odds Ratio, CI = Confidence Interval.

All pooled estimates were derived using the DerSimonian and Laird random-effects model, due to underlying between-study heterogeneity. These findings are illustrated in Fig. 2(a–e) and detailed in Table 3.

**Table 3:** Summary of pooled odd’s ratio for *TNFα 308 G/A* rs1800629 polymorphism with childhood NS risk genetic model.

Genetic models	Sample size (case/controls)	Effect model	OR(95% CI)	95% PI	*P*-Value	Heterogeneity		
						Q	I^2^	*P*_H_
GA + AAvs GG dominant model	628/932	Random	2.57 (1.29 to 5.12)	0.97–6.81	0.0068*	19.61	79.6	0.0006
AAVs GA + GG recessive model	628/932	Random	5.05 (1.48 to 17.23)	0.89–28.65	0.0096*	0.20	33	0.2012
GA vs. GG co-dominant model	628/932	Random	1.86 ( 1.09 to 3.17)	0.88–3.950.88–3.950.88–3.95	0.0219*	11.00	63.7	0.0265
AA vs. GG . co- dominant model	628/932	Random	5.69 ( 1.59 to 20.36)	0.94–34.52	0.0075* 0.73	6.38	37.4	0.1721
A vs. G Allele model	628/932	Random	2.32 ( 1.27to 4.20)	1.00–5.37	0.0056*	20.02	80	0.0005

*- Indicates statistical significance, Q- Cochran Q value, P_H_- P value of heterogeneity; OR- odds ratio; CI- Confidence interval; PI- Prediction Interval, P- value of chi-square test, I^2^ **-** heterogeneity.

## Publication bias

**Fig. 3:**
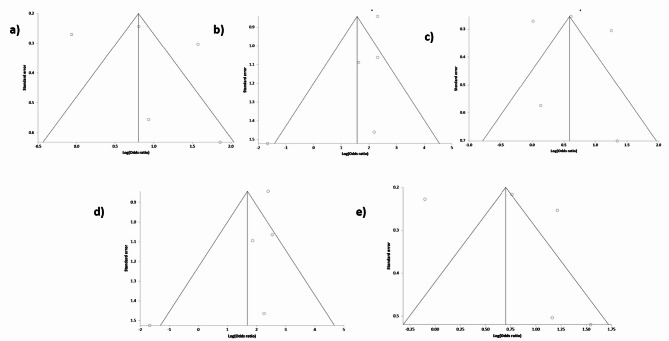
Representative image of Begg’s funnel plot analysis for publication bias. (a) Dominant model (b) recessive model (c) GA vs. GG (d) AA vs. GG, and (e) A vs. G allele for TNFα -308 G/A.

To measure the possibility of publication bias, we utilized Begg’s funnel plot analysis and conducted Egger’s bias test. For TNFα 308 G/A dominant GA + AA vs. GG, heterozygous [GA + GG], and Allele [A vs. G] models, no significant asymmetry was observed in the funnel plots, and Egger’s bias test Fig. 3**(a-e)** did not indicate substantial publication bias in these models. However, in the TNFα 308 G/A polymorphism recessive [AA vs. GA + GG] models and homozygous [AA vs. GG] comparison, notable publication bias was identified, as represented in Table 4.

**Table 4:** Evaluation of publication bias by egger’s test for *TNFα 308 G/A* rs1800629 polymorphisms with NS risk.

Genetic models	Egger’s test *P*-value
Dominant model	0.4833
Recessive model	0.0167*
Heterozygote model	0.8167
Homozygote model	0.0165*
Allele model	0.4833

## Discussion

The exact pathogenesis of nephrotic syndrome remains unclear; however, immune system dysregulation is known to play a pivotal role.Current researches suggested the involvement of a circulating permeability factor derived from dysfunctional T-cells, with various cytokines particularly *TNF-α* proposed as potential contributors^20^. In this study we systematically reviewed 308 G/A SNP in the *TNFα* gene, thoroughly investigating their potential association with the risk of Childhood NS. Following this a meta-analysis carried out from data from five relavent studies, with a specific focus on the *TNFα* 308 G/A rs1800629 polymorphism. Our outcome unveil a noteworthy correlativity between the TNFα -308 G/A polymorphism and the susceptibility to Childhood NS across populations, encompassing individuals in India, Iran, and Egypt. Approximately 50–60% of children diagnosed with SRNS have an unknown cause, while the remaining patients exhibit glomerular podocyte impairment due to a single gene defect^21^.Cytokines pivotal in the development of NS and variants in a single nucleotide within the promoter region of a cytokine gene can impact its expression. This, in turn, can result in modified susceptibility to the disease and varied responses to therapeutic interventions^22^. A pilot study reported Weissbach* et al*.,^23^, persistently elevated* TNFα* levels in steroid-resistant nephrotic syndrome, suggesting its role in steroid non-responsiveness and disease pathogenesis.

Understanding the genetic factors underlying cytokine gene pathogenic mechanisms is valuable for categorization and the formulation of effective treatment strategies, even though these genes have a low prevalence rate. *TNF-α*, specifically, holds a central position in the initiation and advancement of various inflammatory and immunological disorders^24^. Among the Th1 cytokines [*TNF-α*] is a potent immunomodulatory and inflammation-promoting agent closely linked to various pathological disorders. Individuals with active primary Nephrotic Syndrome demonstrated elevated *TNF-α* production levels compared to controls, with notable polymorphism in *TNF-α* production observed during periods of remission^5^. Additionally, *TNF-α* and *IL-1* were positively correlated with proteinuria levels, mesangial hypercellularity, and glomerulosclerosis, providing perceptions into the potential function of *TNF-α* in the pathophysiology of proteinuria and pathological alterations in NS^25^.

Individuals carrying the A allele are believed to exhibit increased TNF-α expression, thereby contributing to heightened inflammatory responses and renal injury^26^.*TNF-α* functions as a pro-inflammatory cytokine crucial for triggering the immune response by activating T and B cells. Transcription of TNF is linked to a polymorphism at position 308, involving a G to A base change in the *TNF-α* promoter^27^. Increased *TNF-α* levels have been observed in plasma and urine of Nephrotic Syndrome patients^28^. Our research also correlates with the above-stated findings, further substantiating the association between the TNF-α 308 G/A (rs1800629) polymorphism and increased susceptibility to childhood Nephrotic Syndrome. By conducting a meta-analysis that pooled data from five independent case-control studies, encompassing 628 NS patients and 932 healthy controls, we identified a significantly increased risk of NS among individuals carrying the A allele or AA genotype of the TNF-α variant. This elevated risk was consistently observed across multiple genetic models, including the allele model (A vs. G), dominant model (GA + AA vs. GG), recessive model (AA vs. GA + GG), heterozygous model (GA vs. GG), and homozygous model (AA vs. GG). The robustness of these associations was further supported by subgroup analysis, which confirmed the consistency of these findings in population-based studies. Collectively, our study reinforces the hypothesis that the A allele in the TNF-α − 308 polymorphism plays a contributory role in the development of childhood Nephrotic Syndrome, likely through enhanced cytokine expression and immune activation. These insights underscore the potential utility of TNF-α − 308 G/A as a genetic biomarker for disease susceptibility and provide a foundation for future research into its functional significance and clinical implications.

## Conclusion

This meta-analysis, which included 1,560 participants (628 cases and 932 controls), shows a strong correlation between the TNFα -308 G/A (rs1800629) polymorphism and the risk of developing childhood nephrotic syndrome. The A allele showed a significant pooled odds ratio of 2.32 (*P* = 0.0056*), suggesting a possible elevated risk. The co-dominant models (AA vs. GG: *P* = 0.0075*; GA vs. GG: *P* = 0.0219*) and the recessive model (*P* = 0.0096*) both showed significant associations, but the dominant model did not reach statistical significance (*P* = 0.068). These results emphasise the clinical significance of TNFα promoter polymorphism as a genetic risk marker and suggest that it may play a part in the pathophysiology of childhood nephrotic syndrome.

In summary, a critical area of interest in pediatric Nephrotic Syndrome centers on understanding the genetic factors that impact the disease’s pathophysiology, progression, and response to treatment, all of which hold significant clinical relevance. In this study, emphasis has been placed on TNF-α as a crucial contributor to the development and progression of diverse inflammatory conditions linked to proteinuria in Nephrotic Syndrome. This research marks a initiative to explore the potential impact of TNF-α promoter gene polymorphisms, specifically the − 308 G/A variant and its related haplotype, on susceptibility to Nephrotic Syndrome.

In summary, the outcomes of our meta-analysis indicate that individuals harboring the at-risk allele of the TNFα 308 G/A polymorphism might face an exaggerated likelihood of developing Childhood Nephrotic Syndrome. Nevertheless, it is crucial to emphasize the importance of conducting more extensive case-control association studies encompassing diverse populations. Such endeavors are essential for gaining deeper insights into the involvement of these genes in the pathogenesis of Childhood Nephrotic Syndrome.

## Limitations

Despite the important findings of this meta-analysis, several limitations must be acknowledged. Firstly, the relatively small number of included studies limits the statistical power and prevents reliable estimation of between-study variance (Tau²), thereby affecting the robustness of the conclusions. The generalizability of the findings is also restricted, as most studies were conducted in Middle Eastern populations, with only one from India, which may not reflect broader ethnic diversity. Moreover, the high level of between-study heterogeneity (I² = 80%) poses challenges to interpreting pooled results and compromises the accuracy of publication bias assessments using Egger’s test, which can yield false-positive outcomes under such conditions. The inability to perform subgroup analyses or meta-regression due to insufficient data further limits the exploration of sources of heterogeneity. Although meta-regression could have provided insight into potential sources of heterogeneity such as gender ratio, mean age, or minor allele frequency this was not feasible due to the small number of studies and inconsistent reporting of these variables. .Additionally, nephrotic syndrome is a multifactorial condition influenced by genetic, environmental, infectious, and psychosocial elements. Since this study is based on aggregated published data, it lacks the ability to adjust for baseline characteristics and potential confounders. The literature search was also restricted to English-language publications, which introduces language bias, and the asymmetry observed in funnel plots may reflect a higher likelihood of positive findings being published over negative results. While these limitations are inherent to retrospective meta-analyses, the consistency of the observed associations supports the relevance of TNFα -308 G/A as a genetic marker in NS, highlighting the need for larger, ethnically diverse studies to confirm these findings.

## Supplementary Information

Below is the link to the electronic supplementary material.

Supplementary Material 1

Supplementary Material 2   

Supplementary Material 3

Supplementary Material 4

Supplementary Material 5

## Data Availability

No datasets were generated or analysed during the current study.
