# Correction: Impact of tumor necrosis factor-alpha gene variant in pediatric nephrotic syndrome: a meta-analysis

**DOI:** 10.1038/s41598-025-22709-5

**Published:** 2025-10-13

**Authors:** Yogalakshmi Venkatachalapathy, Praveenkumar Kochuthakidiyel Suresh, Thendral Hepsibha Balraj, Vettriselvi Venkatesan, Sangeetha Geminiganesan, Indira Bhagam, C. D. Mohana Priya

**Affiliations:** 1 Department of Human Genetics, Sri Ramachandra Institute of Higher Education and Research, Chennai, India; 2 Department of Biochemistry, Ethiraj College for Women, Chennai, India; 3 Kauvery Hospital, Chennai, India; 4 Sri Ramachandra Institute of Higher Education and Research, Chennai, India

Correction to: *Scientific reports* 10.1038/s41598-025-15387-w, published online 14 August 2025

The original version of this Article contained an error in Figure 1, where an incorrect figure was typeset as Figure 1. The incorrect Figure 1 along with its captions is provided below.

**Figure 1:**
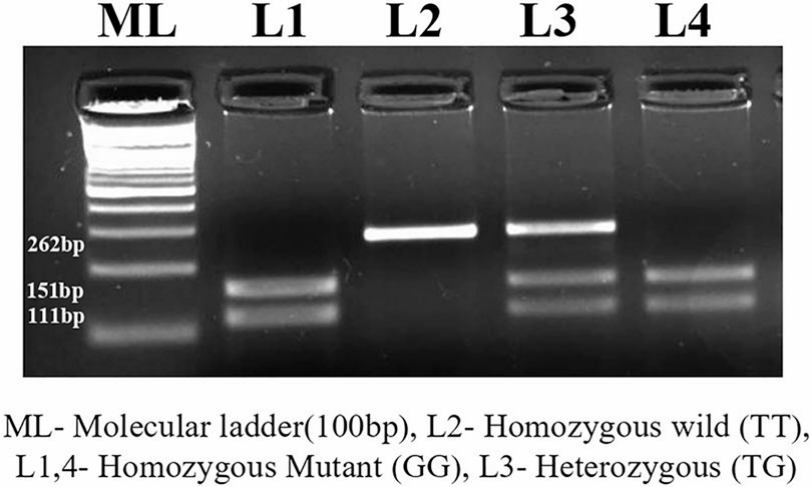
PRISMA flow diagram of studies selected for the meta-analysis.

The original Article has been corrected.

