# Supplementary material for: Impact of tumor necrosis factor-alpha gene variant in pediatric nephrotic syndrome: a meta-analysis
Source: Sci Rep. 2025 Aug 14;15:29797. doi: 10.1038/s41598-025-15387-w (PMC12354666; doi:10.1038/s41598-025-15387-w)
Supplement: Supplementary file 2 — Supplementary Material 2 [file 41598_2025_15387_MOESM2_ESM.doc]

**Association of Cytokine TNF-α gene polymorphism with Childhood Nephrotic Syndrome: a Meta-analysis**

Yogalakshmi Venkatachalapathy^1^, Praveen Kumar Kochuthakidiyel Suresh^1^, Thendral Hepsibha Balraj^2^, Vettriselvi Venkatesan^1^, Sangeetha Geminiganesan^3^, Indira Bhagam^4^, **C.D.Mohana Priya**^1*^

^1^Department of Human Genetics, Sri Ramachandra Institute of Higher Education and Research, Chennai, India, ^2^Department of Biochemistry, Ethiraj College for Women, ^3^ Kauvery Hospital, Chennai, ^4^Faculty of Biomedical Sciences, Sri Ramachandra Institute of Higher Education and Research, Chennai, India

**Supplementary Table S1: Included Studies and Reasons for Inclusion in the Meta-Analysis of TNF-α rs1800629 (G>A) Polymorphism in Childhood NS**

| **S.No** | **Study citation** | **Reason for inclusion** |
| --- | --- | --- |
| 1. | Sadeghi-Bojd S, Hashemi M, Firoozi-Jahanigh M, Rezaei M, Sarani H, Taheri M. Lack of Association Between TNF-alpha rs1800629 (-308G > A) Polymorphism and Nephrotic Syndrome. Iran J Kidney Dis. 2021 Mar;1(2):95-100. PMID: 33764319. | Sufficient information about *TNF*α 308 G/A genotype with same study design |
| 2. | Youssef DM, Amal S, Hussein S, Salah K, Abd El Rahman EA. Tumor necrosis factor alpha gene polymorphisms and haplotypes in Egyptian children with nephrotic syndrome. Cytokine. 2018 Feb 1;102:76-82. | Sufficient information about *TNF*α 308 G/A genotype |
| 3. | Jafar T, Agrawal S, Mahdi AA, Sharma RK, Awasthi S, Agarwal G. Cytokine gene polymorphism in idiopathic nephrotic syndrome children. Indian Journal of Clinical Biochemistry. 2011 Jul;26[3]:296-302. | Sufficient information about *TNF*α 308 G/A genotype |
| 4. | Midan DA, Elhelbawy NG, Ahmedy IA, Noreldin RI. Cytokine gene polymorphism in children with idiopathic nephrotic syndrome. Iranian journal of kidney diseases. 2017 Nov 1;11[6]:414. | Sufficient information about *TNF*α 308 G/A genotype |
| 5. | Madani HA, Bazaraa HM, Rady H. Association of cytokine genes polymorphisms and the response to corticosteroid therapy in children withidiopathic nephrotic syndrome: A pilot study in Egypt. International Research Journal of Medicine and Medical Sciences. 2014;2[4]:84-90. | Sufficient information about *TNF*α 308 G/A genotype |

**Supplementary Table S2: Characteristics of the Included SNP (TNF-α rs1800629)**

| **SNP ID** | **Chromosome** | **Gene** | **Position (GRCh38)** | **Localization** | **Risk Allele** | **Protective Allele** |
| --- | --- | --- | --- | --- | --- | --- |
| rs1800629 | 6 | **TNF** | 6:31543005 | Promoter region | **A** | **G** |
