# Supplementary material for: Impact of tumor necrosis factor-alpha gene variant in pediatric nephrotic syndrome: a meta-analysis
Source: Sci Rep. 2025 Aug 14;15:29797. doi: 10.1038/s41598-025-15387-w (PMC12354666; doi:10.1038/s41598-025-15387-w)
Supplement: Supplementary file 5 — Supplementary Material 5 [file 41598_2025_15387_MOESM5_ESM.docx]

**Supplemtary material**

Supplementary Table S1: Included Studies and Reasons for Inclusion in the Meta-Analysis of TNF-α rs1800629 (G>A) Polymorphism in Childhood NS

Supplementary Table S2: Characteristics of the Included SNP (TNF-α rs1800629)

PRISMA 2020 Checklist document

PRISMA 2020 extension for abstracts checklist document

Supplementary excel- Genotyping data of the rs1800629 SNP of the included studies
